# AETHER: Video-Based Smartphone Free Flap Monitoring Using Spatial Phase Coherence and Reference-Corrected Augmented Imaging

**DOI:** 10.34133/csbj.0137

**Published:** 2026-06-18

**Authors:** Christoph Wallner, Alexander Fiedler, Sonja Verena Schmidt, Felix Reinkemeier, Flemming Puscz, Alexander Sogorski, Marcus Lehnhardt, Marius Drysch

**Affiliations:** Department of Plastic and Reconstructive Surgery, BG University Hospital Bergmannsheil Bochum, Ruhr-University Bochum, Bochum, Germany.

## Abstract

**Background:** Postoperative free flap surveillance relies on clinical inspection or hardware-dependent adjuncts. Remote photoplethysmography offers a contactless alternative, but motion, lighting variation, specular reflection, and camera autoexposure can mimic perfusion signals. AI-Enhanced Transcutaneous Hemodynamic Evaluation for Reconstructive surgery (AETHER) combines per-patch spatial phase coherence, in-image reference correction, and augmented overlay visualization. **Methods:** We retrospectively analyzed bedside smartphone videos of 25 consecutive anterolateral thigh (ALT) free flaps treated between November 2025 and April 2026. No patient-level demographic, comorbidity, clinical-suspicion, or bedside-examination data entered the algorithm; AETHER was evaluated as a covariate-free, video-only signal-processing proof of concept. Flap and nonperfused reference regions of interest, each divided into 100 subregions, underwent plane-orthogonal-to-skin-based pulse extraction, band-pass filtering, and sliding-window correlation with a global mean reference. The Global Coherence Score (GCS) was defined as the percentage of synchronous subregions; corrected GCS was flap GCS minus reference GCS. Compromise was defined as clinically indicated surgical take-back with intraoperative confirmation of arterial compromise. **Results:** Six flaps had arterial compromise, and 19 were vital. Raw flap GCS and reference GCS did not separate groups (area under the receiver operating characteristic curve 0.62 and 0.36, respectively). Corrected GCS showed strong observed in-sample case-level separation (area under the receiver operating characteristic curve 0.96, 95% confidence interval 0.87 to 1.00). At an exploratory 17% cutoff, sensitivity was 0.83 and specificity 0.89; predictive values were prevalence-dependent. **Conclusions:** AETHER identified a video-derived perfusion-associated signal from routine smartphone video alone. These findings support covariate-free signal extraction but not recording-level diagnostic performance, lead time, or generalizability. Prospective validation is required before clinical use.

## Introduction

Vascular compromise after free tissue transfer is a race against time. Salvage rates exceed 80% within the first 12 postoperative hours but fall below 1 in 3 after 72 h, and a single take-back can raise hospitalization costs by approximately 94% [1–4]. Large-series analyses across more than 2,000 head-and-neck free flaps report a total necrosis rate of approximately 4% with the majority of thrombotic events occurring within the first 3 postoperative days, and salvage of arterial thrombosis remains markedly lower than salvage of venous compromise [5]. Continuous, low-burden surveillance is therefore essential, yet every available option carries a trade-off.

Clinical inspection (skin color, temperature, capillary refill, turgor, and pin prick) is universal but subjective, observer-dependent, and labor-intensive, and modern reductions in resident working hours have eroded the historically continuous bedside coverage on which it depended [6,7]. Implantable Doppler probes provide continuous pedicle-level information but are invasive and have historically been associated with high false-alarm rates [8]; more recent reports describe substantially improved positive predictive values (PPVs) with current devices, and we contextualize the older figures accordingly. Indocyanine green (ICG) fluorescence angiography offers high spatial resolution but is episodic, dye-dependent, and reliant on dedicated hardware [9]. Near-infrared spectroscopy (NIRS) is continuous but hardware-dependent and cost-intensive [10]. Laser Doppler flowmetry (LDF) provides continuous local microcirculatory information but is contact-dependent, susceptible to sensor detachment, and limited to a single point of measurement [11]. Photoplethysmography (PPG)—the optical detection of capillary blood-volume changes—has decades of clinical history as a contact technology in pulse oximetry [12,13]; recent intraoperative remote photoplethysmography (rPPG) work has demonstrated that the same principle can be recovered from standard video in anastomosed fasciocutaneous flaps [14], and imaging photoplethysmography in head-and-neck reconstruction has shown that spatial information is essential to capture heterogeneous compromise [15].

In practice, however, the optical environment of the ward and the operating theatre is hostile: Specular reflections, transmitted respiratory motion under mechanical ventilation, ambient illumination drift, camera autoexposure and white-balance regulation, dressings, and operator handling all generate fluctuations within the cardiac frequency band that single-region or amplitude-based pipelines cannot distinguish from genuine perfusion [16]. The chromatic basis of rPPG—capillary hemoglobin volume modulations of diffuse reflectance, predominantly in the green channel—limits its interrogation depth to the superficial dermis (~0.5 to 1.0 mm), and the resulting signal does not directly probe anastomotic patency or deep flap perfusion. PPG morphology is additionally influenced by vessel stiffness and systemic hemodynamics and changes with age, irradiation, and postsurgical tissue remodeling [17].

Three design principles motivate AI-Enhanced Transcutaneous Hemodynamic Evaluation for Reconstructive surgery (AETHER). First, perfusion is quantified spatially rather than as a single trace: The flap is decomposed into 100 subregions whose pulse signals are evaluated for spatial phase coherence. A single noisy patch reduces the coherence count by 1 of 100 rather than corrupting the global metric, conferring robustness against the local artifacts that have limited amplitude-based pipelines. Second, an in-image nonperfused reference region is recorded simultaneously and used to correct for shared environmental artifacts on a common-mode basis, exploiting the fact that flap and reference are exposed to identical optical conditions by construction. Third, the per-patch coherence pattern is rendered as an augmented overlay on the original image, providing a spatially resolved bedside readout that integrates with routine workflow.

We deliberately chose an interpretable signal-processing approach rather than a deep-learning model. In a pilot cohort of 25 flaps with 6 events, training a black-box classifier would be a textbook example of insufficient data. Interpretable signal processing supports transparent inspection of failure modes, computational lightness sufficient for on-device inference on commodity smartphone hardware, deployability without graphics processing unit resources, and auditability at the bedside. Artificial intelligence (AI)-based components—automated segmentation, quality control, automated region of interest (ROI) selection, and temporal prediction—are valuable complements that we identify as future work.

By design, AETHER does not incorporate patient-level covariates or clinical assessments. This distinguishes the present work from a clinical risk model. The technical question is narrower but fundamental: whether routine video alone contains sufficient optical structure to separate ultimately vital flaps from cases with surgically confirmed arterial compromise after correction for in-image common-mode artifacts. Because age, sex, body mass index, comorbidities, prior radiotherapy, smoking status, laboratory values, and bedside clinical impression do not enter the algorithm, any observed separation cannot be attributed to demographic risk stratification or to clinician-provided labels embedded in the input. This does not eliminate selection bias at the case level, but it isolates the optical signal-processing claim from the multivariable confounders that would dominate a clinical risk model.

AETHER is not proposed as a replacement for clinical monitoring. It is designed as an objective, contactless, low-burden spot-check adjunct. Here, we evaluate AETHER in a retrospective video-only proof-of-concept cohort of 25 consecutive anterolateral thigh (ALT) free flaps and report the case-level signal separation achieved by the corrected Global Coherence Score (GCS) together with its limitations, prevalence dependence, and the prospective validation requirements that must be met before any clinical threshold can be considered established.

## Methods

### Study design

We retrospectively analyzed consecutive adult patients undergoing ALT free flap reconstruction at a tertiary university hospital between November 2025 and April 2026 for whom bedside smartphone video material was available. Each recording included the flap and an adjacent nonperfused area (surgical drape or gauze) within the same field of view. Compromise was defined as surgical take-back with intraoperative confirmation of arterial compromise; flaps with an uncomplicated postoperative course and confirmed clinical viability at discharge were classified as vital. The patient-flow diagram is shown in Fig. 1. The study was approved by the institutional review board (Ärztekammer Westfalen-Lippe, no. 2026-037-f-S); informed consent was waived owing to the retrospective design and exclusive use of fully anonymized material.

**Fig. 1.**
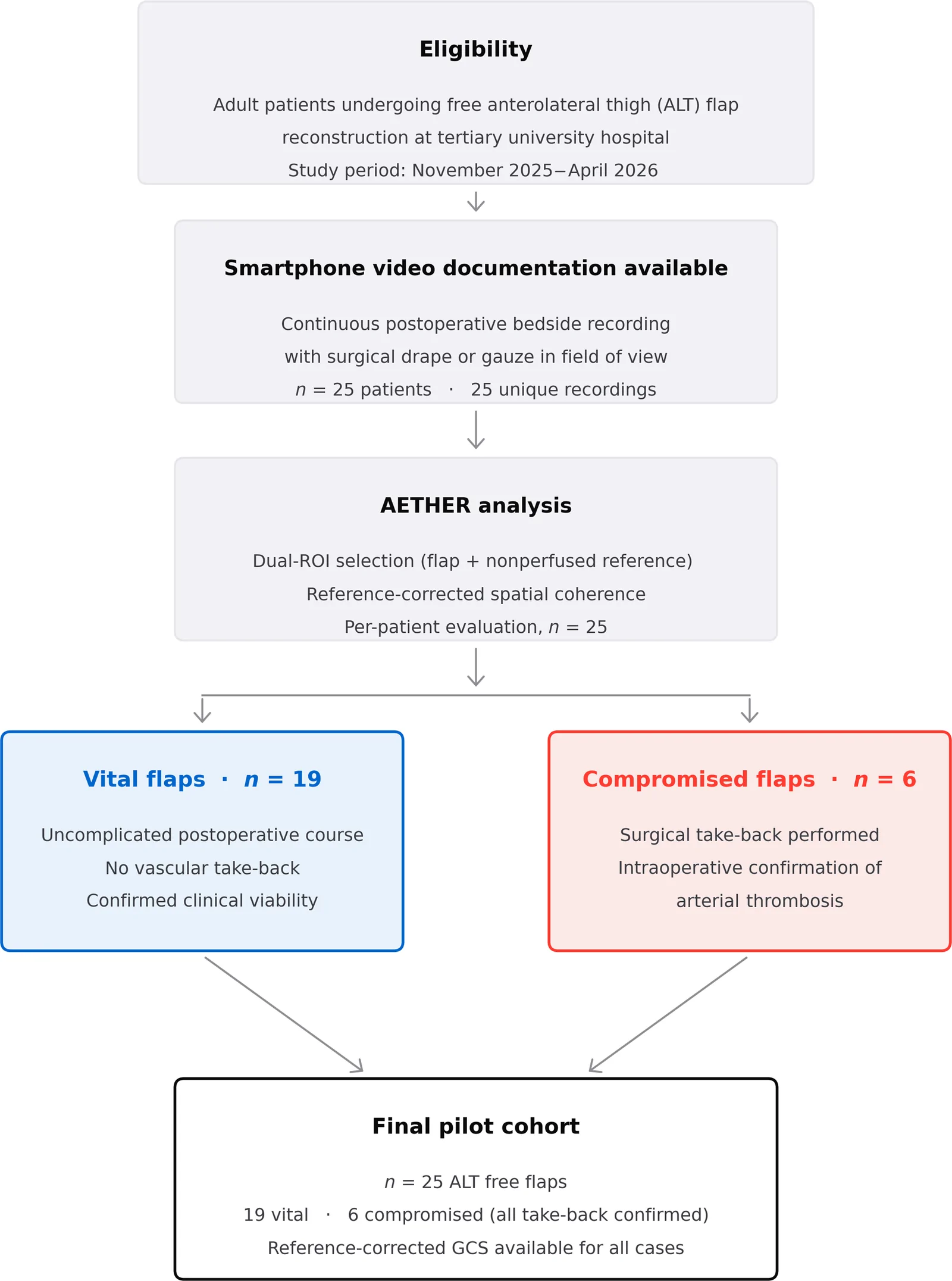
Patient flow. Consolidated Standards of Reporting Trials (CONSORT)-style flow diagram of the retrospective cohort. The analytic cohort comprised all consecutive anterolateral thigh (ALT) free flap cases for which bedside smartphone video material met predefined technical criteria—flap and a nonperfused area in the same field of view, and a contiguous artifact-free segment of at least 10 s (*n* = 25). Outcomes were stratified into 19 vital flaps (uncomplicated postoperative course, no take-back) and 6 compromised flaps (surgical take-back with intraoperative confirmation of arterial compromise).

The approved analytic dataset comprised anonymized bedside video material, derived technical signal outputs, flap type, outcome category, and intraoperatively confirmed mechanism in revised cases. Patient-level demographic, comorbidity, and temporal clinical metadata were not part of the approved dataset and were not reconstructed or imputed. Accordingly, AETHER was evaluated as a covariate-free, video-only signal-processing method: No clinical covariate enters the algorithm at any stage. This design allows assessment of whether the optical video signal itself contains discriminative information between ultimately vital and surgically confirmed arterial-compromise cases, but it precludes assessment of demographic effect modification, clinical generalizability, and recording-level lead time. These questions require a prospectively designed study under an extended ethics protocol and are addressed in the “Outlook and future work” section.

### Cohort

The analytic cohort comprised all consecutive ALT free flap cases for which bedside smartphone video material met predefined technical criteria—flap and a nonperfused area in the same field of view, and a contiguous artifact-free segment of at least 10 s (*n* = 25 over a 6-month window). Because exhaustive identification of all ALT free flaps performed during the period was beyond the scope of the anonymized analytic dataset, we do not report an institutional event rate; the cohort is described as the subset of ALT cases with adequate bedside video documentation.

### Video acquisition

Recordings were obtained at the postoperative bedside under routine ambient lighting using consumer-grade smartphone cameras (Apple iPhone, multiple generations; Samsung Galaxy series; rear-facing wide-angle module, 1,920 × 1,080 pixels at 30 frames per second, H.264 encoding). The analysis interval was selected as the first contiguous segment of at least 10 s free of operator intervention, unstable framing, specular saturation, and gross motion; recordings exceeding this window were truncated to a maximum of 60 s (≈ 1,800 frames at 30 fps) to bound computational cost. The median analysis interval was 14.0 s (range 10.0 to 22.5).

### AETHER system

The full processing pipeline is summarized in Fig. 2. After loading the analysis interval, 2 regions of interest were defined sequentially in the first frame: a flap ROI covering the cutaneous monitor island and a reference ROI placed on a nonperfused area (typically a fold of surgical drape adjacent to the flap, of comparable area and within the same focal plane). The 2 ROIs were processed by an identical signal chain.

**Fig. 2.**
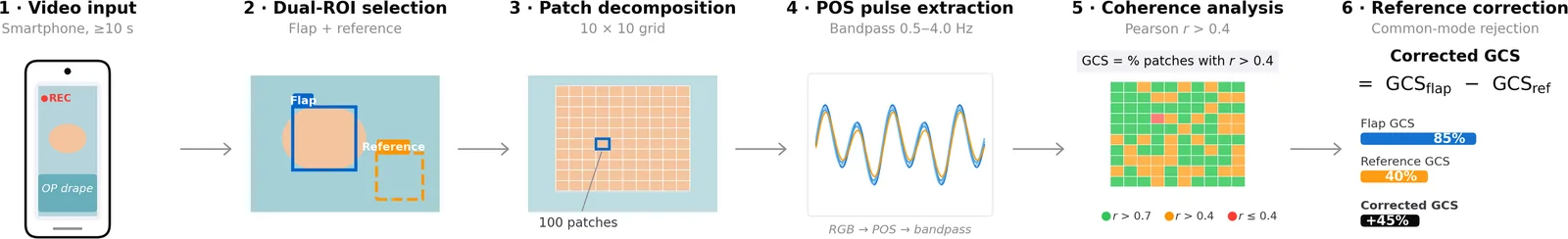
The AETHER pipeline. Six processing steps from smartphone video to reference-corrected coherence score. (1) Postoperative bedside video acquisition with the flap and a nonperfused area in the same field of view. (2) Sequential dual-region of interest (ROI) selection: flap (solid blue) and reference (dashed amber). (3) Each ROI is decomposed into a 10 × 10 grid of 100 subregions. (4) Per-patch pulse signals are extracted with the plane-orthogonal-to-skin (POS) algorithm and band-pass-filtered (0.5 to 4.0 Hz). (5) The Global Coherence Score (GCS) is the percentage of subregions correlated (Pearson *r* > 0.4) with the per-recording global mean reference, computed independently for the flap and reference ROIs. (6) The corrected GCS is the difference between the 2 scores and serves as the primary exploratory diagnostic metric. All panels rendered at 300 dpi.

Frame-wise stabilization was performed using the pyramidal Lucas–Kanade optical-flow algorithm with 3 pyramid levels and a 21 × 21 search window, tracking up to 200 Shi–Tomasi corner features inside each ROI to compensate for transmitted physiologic motion (ventilation, cardiac wall movement, and microtremor). The global affine transform recovered at each frame was applied as an inverse warp before any further processing. Residual motion that affects flap and reference differently is not corrected for and remains a failure mode.

Each stabilized ROI was decomposed into a 10 × 10 spatial grid yielding 100 nonoverlapping subregions. For each subregion, the spatially averaged RGB time series was projected onto the plane orthogonal to the standard skin-tone vector following the plane-orthogonal-to-skin algorithm of Wang et al. [18], which suppresses intensity-related artifacts arising from specular drift and global luminance changes while preserving the chromatic color modulations induced by capillary perfusion. The resulting per-patch pulse traces were filtered with a fourth-order zero-phase Butterworth bandpass between 0.5 and 4.0 Hz, encompassing the physiological cardiac range from 30 to 240 bpm. A global mean reference signal was constructed by averaging the 100 filtered traces; this aggregate exploits the law of large numbers—spatially uncorrelated noise cancels in the mean while a synchronous cardiac component is reinforced—and serves as the per-recording template against which individual subregions are compared. Heart rate was inferred from the dominant frequency of this global mean reference within the cardiac band.

Spatial phase coherence was quantified by the windowed Pearson correlation coefficient between each subregion’s filtered pulse trace and the global mean reference, computed over a sliding window of 90 frames (3 s at 30 fps) advanced one frame at a time. Subregions whose correlation coefficient at a given time point exceeded *r* > 0.4 were classified as synchronous, a threshold consistent with conventional moderate-correlation criteria in biomedical signal analysis. The GCS was defined as the percentage of synchronous subregions and was computed independently for the flap ROI and for the reference ROI. The recording-level GCS reported throughout this study is the median of the per-frame GCS time series, providing robustness against transient stabilization failures or short subwindow dropouts.

### Terminology of reference signals

Two reference concepts are used. The internal coherence template denotes the global mean signal computed within a given ROI from its 100 filtered patch traces; it is used only to quantify spatial synchrony within that ROI. The external reference ROI denotes the simultaneously recorded nonperfused surgical drape or gauze region; its GCS estimates the in-image common-mode artifact baseline. The corrected GCS is defined as flap ROI GCS minus external reference ROI GCS.

### Reference correction

Because the flap and reference ROIs are recorded simultaneously by the same camera, they share illumination drift, sensor autoadjustment, transmitted motion, and specular reflection. These common-mode artifacts may produce coherent optical fluctuations within the cardiac frequency band even in nonperfused material. We therefore computed GCS independently for the flap ROI and the nonperfused reference ROI using the identical processing pipeline and defined:

CorrectedGCS=GCS_flap−GCS_reference. (1)

This value represents the surplus spatial coherence within the flap beyond the in-image artifact baseline. Values near zero indicate that apparent flap coherence is not distinguishable from the simultaneously measured background artifact coherence.

### ROI selection—Current limitations

ROI placement (flap and reference) is performed manually in the current implementation and is operator-dependent. We did not perform formal inter- or intraoperator reproducibility testing in this retrospective cohort, and we identify this as a major methodological limitation. Heterogeneous compromise, partial necrosis with preservation of a viable margin, and subsegmental ischemia may be missed if ROI placement is not representative of the affected territory. Required next steps are full-surface ROI coverage (rather than a single bounding region), automated semantic segmentation of flap and reference, inter- and intrarater reproducibility testing on a fixed set of recordings, and a standardized acquisition protocol; these are detailed in the “Outlook and future work” section.

### Augmented visualization

To support bedside interpretability, AETHER overlays per-patch coherence on the original image. Each of the 100 flap subregions is color-coded by its sliding-window correlation against the global mean reference (green *r* > 0.7, amber *r* > 0.4, red *r* ≤ 0.4) and rendered alongside a real-time time series of the flap and reference scores.

### Statistical analysis

All analyses are exploratory. The cohort is small (*n* = 25), and the number of events is 6. Group comparisons used the 2-sided Mann–Whitney U test; results are reported as medians and interquartile ranges (IQRs). With *n* = 19 versus *n* = 6, this test is considerably underpowered for a definitive inference: The obtained *P* value reflects the magnitude of separation in this specific cohort, not the reliability with which the test would detect this effect in a new sample. Case-level discrimination is reported as the area under the receiver operating characteristic curve (AUC) with 95% confidence intervals from nonparametric bootstrap (5,000 iterations). The Youden-derived operating point was identified on the same cohort and is therefore prone to optimism; we describe it explicitly as an exploratory working threshold rather than a validated cutoff. Sensitivity, specificity, PPV, and negative predictive value (NPV) are reported with exact 95% Clopper–Pearson confidence intervals. Because PPV and NPV are prevalence-dependent, we additionally report Bayes-projected PPV/NPV at a 5% background prevalence, computed from the point estimates of sensitivity and specificity. Bootstrap confidence intervals on AUC reflect resampling uncertainty within the same cohort and do not substitute for external validation. Per-recording corrected GCS values are provided in Table 1. Analyses were performed in Python 3.11 (NumPy, SciPy, and scikit-learn).

**Table 1.** Cohort summary and exploratory diagnostic performance. Group-level summary of the analytic cohort (*n* = 25). All flaps were ALT free flaps; in all 6 compromised cases, the surgically documented mechanism was arterial compromise. Distributions are reported as median (interquartile range; full range). Sensitivity, specificity, PPV, and NPV are reported with exact 95% Clopper–Pearson confidence intervals; AUC with 95% bootstrap CI (5,000 iterations). All metrics are in-sample and exploratory. The approved anonymized video-analysis dataset did not include patient-level demographic or comorbidity variables.

Variable	Vital (*n* = 19)	Arterial compromise (*n* = 6)	Comparison / metric
Flap type	ALT (19/19)	ALT (6/6)	—
Surgical revision / take-back	0 / 19	6 / 6	—
Intraoperative mechanism (revised cases)	n/a	Arterial compromise (6/6)	—
Analysis interval (s)	14.0 (10.0–22.5)	14.0 (10.0–22.5)	Median (range); cohort-level
Raw flap GCS (%)	73 (50–95; 30–98)	62 (49–69; 43–88)	AUC 0.62 (0.37–0.85); *P* = 0.39
Reference GCS (%)	40 (33–58)	59 (36–77)	AUC 0.36 (0.05–0.71); *P* = 0.32
Corrected GCS (%)	25 (19–38; 10–48)	2 (−2 to +13; −6 to +17)	AUC 0.96 (0.87–1.00); *P* < 0.001
Classification at 17% cutoff: above (vital call)	17 / 19	1 / 6	—
Classification at 17% cutoff: at or below (compromise call)	2 / 19	5 / 6	—
Sensitivity	—	5 / 6 = 0.83	95% CI 0.36–1.00
Specificity	17 / 19 = 0.89	—	95% CI 0.67–0.99
PPV at within-cohort prevalence 24%	—	5 / 7 = 0.71	95% CI 0.29–0.96
NPV at within-cohort prevalence 24%	17 / 18 = 0.94	—	95% CI 0.73–1.00
Bayes-projected PPV at 5% prevalence	—	≈ 0.29	Sens 0.83, Spec 0.89
Bayes-projected NPV at 5% prevalence	≈ 0.99	—	Sens 0.83, Spec 0.89
Final flap outcome at discharge	Viable (19/19)	—	—

ALT, anterolateral thigh; GCS, Global Coherence Score (percentage of synchronous subregions); PPV, positive predictive value; NPV, negative predictive value; AUC, area under the receiver operating characteristic curve; CI, confidence interval

## Results

### Cohort

Twenty-five consecutive ALT free flaps were analyzed. Six of 25 (24%) were surgically revised with intraoperative confirmation of arterial compromise; 19 (76%) had an uncomplicated postoperative course and were classified as vital at discharge (Fig. 1). All 25 recordings completed the AETHER pipeline without technical failure, stabilization breakdown, or analytic exclusion. The median analysis interval was 14.0 s (range 10.0 to 22.5).

The 24% event proportion is a within-cohort rate and is not the institutional free-flap compromise rate, which in published large series ranges from 2% to 8% [5,14]. The within-cohort rate reflects enrichment of cases for which bedside video documentation was available. PPV and NPV reported below are conditional on this within-cohort prevalence; prevalence-adjusted projections are reported alongside the cohort-level estimates.

In all 6 revised cases, the intraoperatively documented mechanism was arterial compromise. Venous congestion, partial flap compromise, subsegmental ischemia, and clinically suspicious but ultimately viable flaps were not represented in this cohort and are addressed in the “Limitations” and “Outlook and future work” sections.

### Raw flap GCS does not separate the groups

Raw flap GCS distributions overlapped substantially across outcomes (Fig. 3A). Vital flaps showed a median of 73% (IQR 50 to 95, full range 30 to 98), and compromised flaps a median of 62% (IQR 49 to 69, full range 43 to 88). The group difference did not reach statistical significance (Mann–Whitney U = 73, *P* = 0.39). Receiver operating characteristic analysis confirmed nondiscrimination, with an AUC of 0.62 (95% bootstrap confidence interval [CI] 0.37 to 0.85; Fig. 4). Eleven of 19 vital flaps fell within the raw-GCS range observed in compromised flaps, and all 6 compromised flaps fell within the range observed in vital flaps. No threshold on the raw flap GCS scale could have separated the 2 groups; at the most favorable in-sample cutoff of 70%, 2 compromised flaps would have been missed, and 9 vital flaps would have been falsely flagged.

**Fig. 3.**
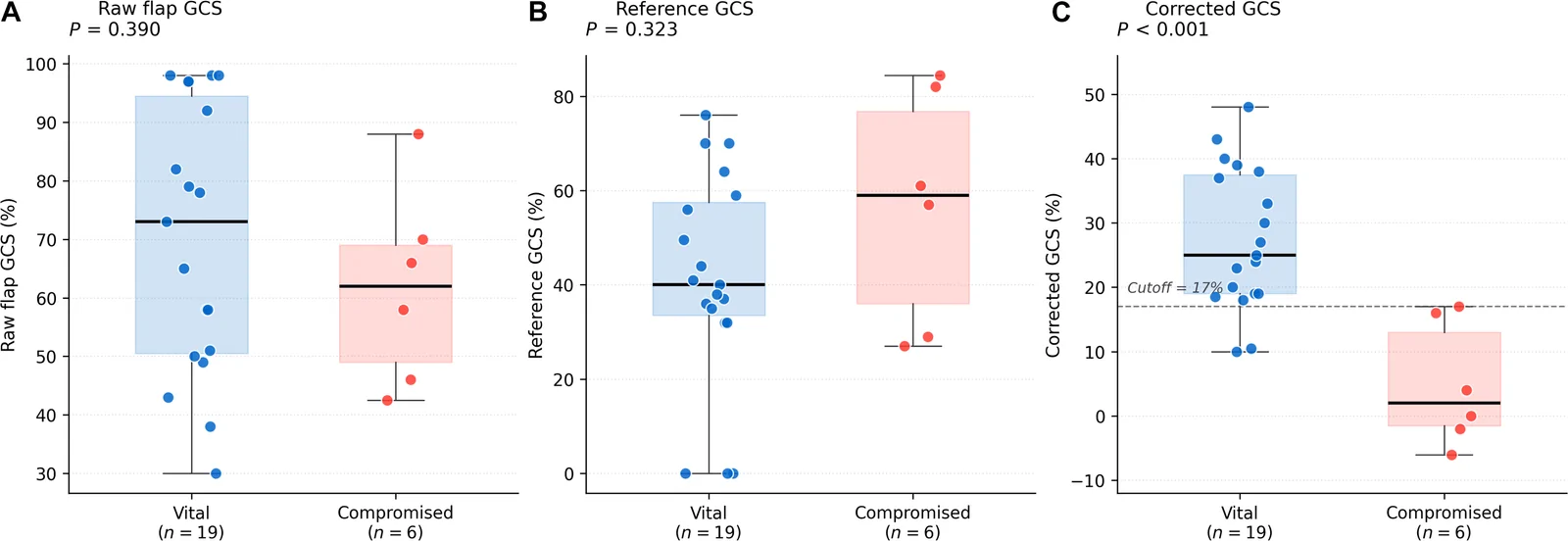
Group separation across coherence measures. Boxplots with overlaid individual measurements stratified by clinical outcome (*n* = 19 vital, *n* = 6 compromised) for (A) raw flap Global Coherence Score (GCS), (B) reference GCS, and (C) corrected GCS. Boxes denote median and interquartile range (IQR); whiskers indicate 1.5 × IQR; circles represent individual flaps. *P* values are derived from 2-sided Mann–Whitney U tests and are exploratory; with *n* = 19 versus *n* = 6 the test is considerably underpowered for a definitive inference. Vital and compromised distributions overlap substantially on raw flap and reference scores; the corrected GCS (flap GCS minus reference GCS) shows strong in-sample discrimination in this small cohort. Dashed line in (C): Youden-derived exploratory working cutoff at 17%, prone to optimism and requiring prospective external validation.

**Fig. 4.**
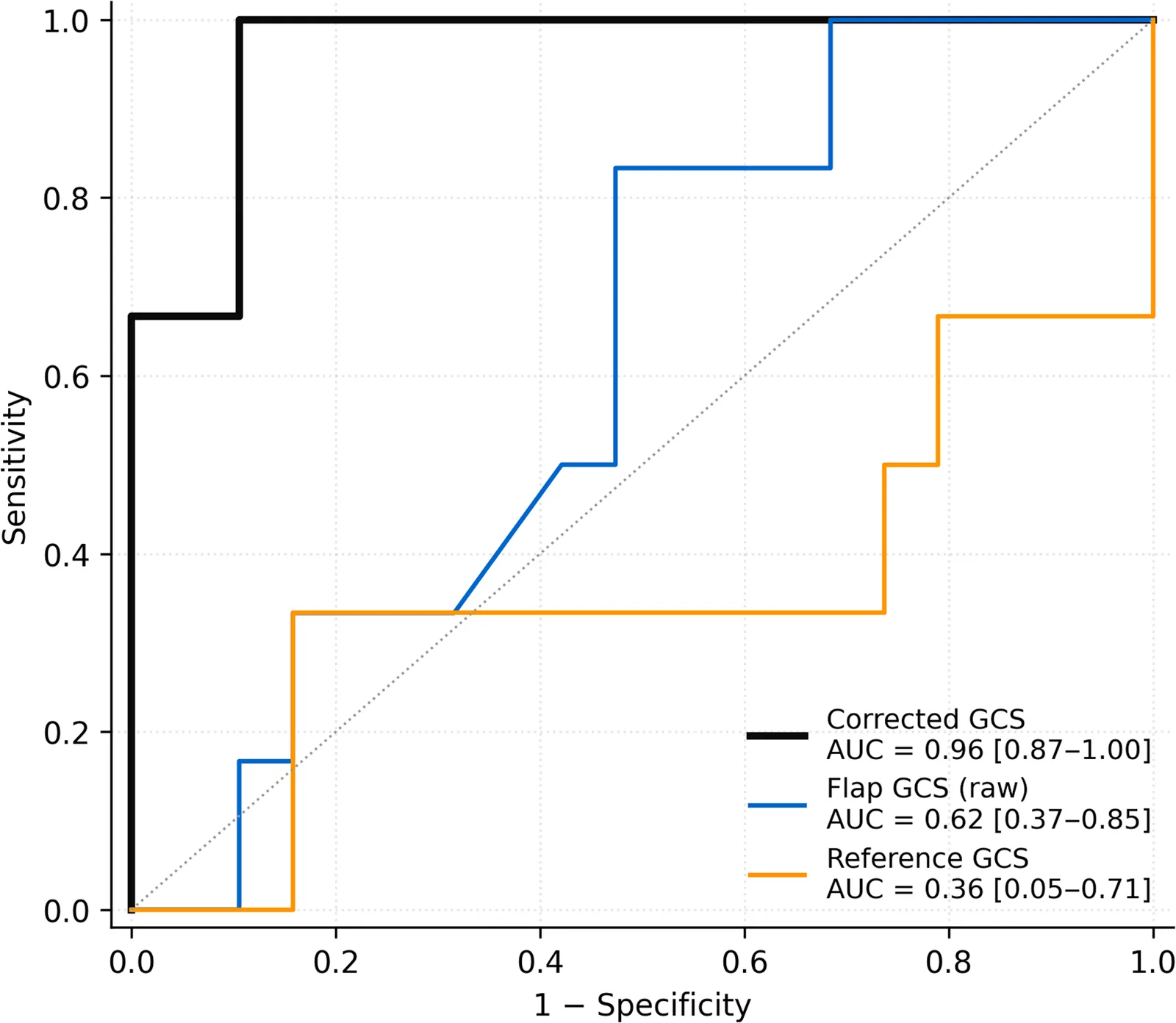
Receiver operating characteristic (ROC) curves for compromise detection. ROC curves for the 3 candidate metrics. Corrected Global Coherence Score (GCS): area under the receiver operating characteristic curve (AUC) 0.96 (95% bootstrap confidence interval [CI] 0.87 to 1.00). Raw flap GCS: AUC 0.62 (95% CI 0.37 to 0.85). Reference GCS: AUC 0.36 (95% CI 0.05 to 0.71). Diagonal: chance performance. All AUC values are in-sample; bootstrap intervals reflect resampling uncertainty within the same cohort and do not substitute for external validation.

### The reference region reveals a substantial artifact baseline

The reference GCS, recorded simultaneously in nonperfused tissue immediately adjacent to each flap, was likewise nondiscriminative (Fig. 3B). Vital recordings showed a median reference GCS of 40% (IQR 33 to 58), compromised recordings 59% (IQR 36 to 77); Mann–Whitney U = 38, *P* = 0.32, AUC 0.36 (95% CI 0.05 to 0.71). Nonperfused drape or gauze regions reached coherence scores as high as 84%. These surfaces contain no microvasculature and cannot generate physiologic pulsatility, yet they produced coherence scores indistinguishable from those of vital flaps. This direct measurement establishes that the artifact baseline is substantial, case-dependent, and embedded within the same frequency band as the cardiac signal and that any rPPG analysis relying on an absolute threshold of flap-only coherence is structurally exposed to systematic confounding.

### Reference correction restores in-sample discrimination

Subtraction of the simultaneously acquired reference score produced a markedly improved, near-disjoint separation in this small exploratory cohort (Fig. 3C). Vital flaps showed a corrected GCS of 25% (IQR 19 to 38, full range 10 to 48), compromised flaps 2% (IQR −2 to +13, full range −6 to +17); Mann–Whitney U = 110, *P* < 0.001. In-sample case-level separation rose to AUC 0.96 (95% bootstrap CI 0.87 to 1.00; Fig. 4), corresponding to a gain of ΔAUC = 0.34 over the raw flap GCS. The improvement is qualitative as well as quantitative: The 2 compromised flaps with the highest raw flap scores in their group (88% and 70%), which would have been the most confidently misclassified by any flap-only threshold, were correctly identified by the corrected score (4% and 0%, respectively), supporting the mechanistic interpretation that the discriminative information is contained in the difference between the 2 regions and not in either region alone. These operating characteristics are exploratory and describe the present enriched analytic cohort; they should not be interpreted as validated clinical diagnostic performance.

### Operating point and misclassifications

On the basis of the observed distribution, corrected GCS values below approximately 10% appeared consistently concerning, values above 17% were generally reassuring, and values between 10% and 17% represented an indeterminate zone requiring repeat acquisition and clinical adjudication. At the Youden-derived exploratory cutoff of 17%, sensitivity was 0.83 (5/6, 95% Clopper–Pearson CI 0.36 to 1.00) and specificity 0.89 (17/19, 95% CI 0.67 to 0.99); positive predictive value was 0.71 (5/7, 95% CI 0.29 to 0.96) and NPV 0.94 (17/18, 95% CI 0.73 to 1.00) at the within-cohort prevalence of 24%. The Clopper–Pearson interval for sensitivity is wide, reflecting the small event count, and the cutoff was derived from the same cohort and is therefore prone to optimism; the cutoff must be regarded as exploratory and requires prospective external validation.

To illustrate the prevalence dependence of the predictive values, we computed Bayes-projected PPV and NPV at a 5% background prevalence using the observed point estimates of sensitivity (0.83) and specificity (0.89). At a 5% prevalence, the projected PPV falls to approximately 0.29, and the NPV rises to approximately 0.99. In other words, at realistic background event rates, the corrected GCS would be informative principally as a rule-out test, with high NPV and limited positive predictive value; this prevalence dependence is a structural property of the system and must be accounted for in any clinical deployment.

Three cases were misclassified, all clustered within 7 percentage points of the threshold. A single false negative at corrected GCS = +17% (compromised flap with partially preserved pulsatility, flap GCS 46%, reference GCS 29%) cannot be unambiguously attributed to genuine partial perfusion or to marginal acquisition signal-to-noise; we discuss the interpretation of such intermediate cases in the Discussion. Two false positives occurred at corrected GCS values of +10.5% and +10.0% (vital flaps with unusually low overall acquisition signal to noise). Outside the +10% to +17% window, all 22 remaining flaps were classified correctly, defining a narrow exploratory gray zone.

### Augmented visualization in 2 representative cases

Two representative cases illustrate the bedside output of the integrated system (Fig. 5). In the vital flap, the augmented overlay rendered a predominantly green spatial coherence pattern across the flap surface; in the monitoring timeline, the flap signal remained sustainedly above the reference baseline throughout the recording, with the 2 curves following independent trajectories (flap GCS 95%, reference GCS 72%, corrected +23%; classified vital). In the compromised flap with surgically confirmed arterial compromise at take-back, the raw flap GCS of 85% with 96 of 100 synchronous subregions would, in isolation, have suggested healthy perfusion. The discriminative finding lay not in the absolute flap value but in the parallel evolution of the 2 timelines: The flap and reference curves rose, fell, and plateaued together, indicating that both regions were responding to the same shared optical environment rather than to independent physiologic sources. The corrected GCS of 5% correctly identified the compromise. Snapshot frames in Fig. 5 were extracted near the midpoint of each analysis interval.

**Fig. 5.**
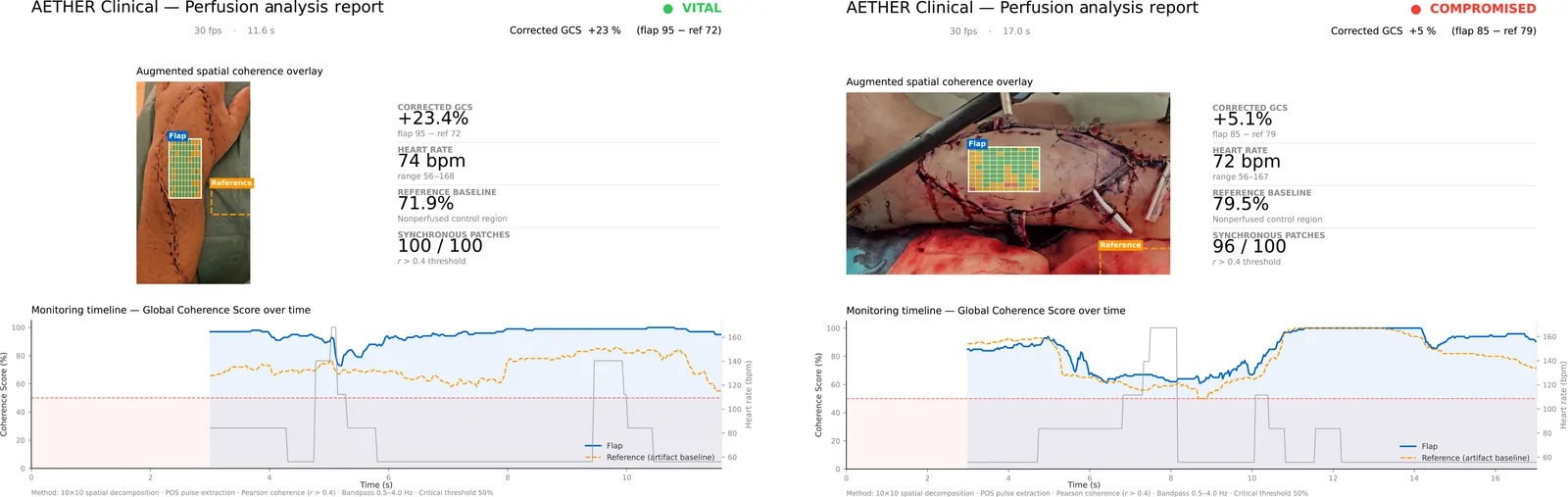
Augmented visualization in 2 representative cases. (Left) Vital anterolateral thigh (ALT) free flap. The augmented spatial coherence overlay shows a predominantly green pattern across the flap surface, with 100/100 subregions classified as synchronous. In the monitoring timeline, the flap signal (blue) is decoupled from the reference baseline (amber dashed), remaining sustainedly above it throughout the recording and following an independent trajectory. Flap Global Coherence Score (GCS) 95%, reference GCS 72%, corrected GCS +23%; classified vital. (Right) Compromised ALT free flap with surgically confirmed arterial compromise at take-back. Raw metrics are reassuring: flap GCS 85% with 96/100 synchronous subregions. The diagnostic finding lies not in the absolute flap value but in the coupled evolution of the 2 timelines: Flap and reference curves rise, fall, and plateau in near-perfect synchrony. The simultaneously recorded reference region on the surgical drape scored 79%, and the corrected GCS of 5% correctly identified the compromise. Snapshot frames extracted near the midpoint of each analysis interval. This case would have been a false negative under any flap-only analysis.

## Discussion

### Principal findings

We present AETHER, a video-based smartphone system for free flap monitoring that integrates spatial phase coherence analysis, in-image reference correction, and augmented visualization in a single end-to-end pipeline. In a retrospective video-only proof-of-concept cohort of 25 consecutive ALT free flaps, the corrected GCS showed strong in-sample case-level separation between ultimately vital flaps and flaps with surgically confirmed arterial compromise (AUC 0.96, 95% bootstrap CI 0.87 to 1.00), corresponding to a sensitivity of 0.83 and a specificity of 0.89 at an exploratory working cutoff of 17%. Neither raw flap coherence (AUC 0.62) nor reference coherence alone (AUC 0.36) was informative. The discriminative signal emerged only from the integration of the 3 system components. These findings are descriptive and exploratory and describe the present enriched analytic cohort; they do not constitute validated recording-level diagnostic performance.

### Mechanistic interpretation

A central observation is that surgical drape and gauze, surfaces that contain no microvasculature, produced spatial coherence scores indistinguishable from those of vital flaps and reaching values as high as 84%. This finding has direct mechanistic implications. The cardiac frequency band (0.5 to 4.0 Hz) is not a private channel reserved for hemoglobin-volume changes; it is also occupied by transmitted respiratory motion under positive-pressure ventilation, by quasi-periodic specular reflections that follow patient and operator micromotion, and by the regulatory loops of consumer-grade autoexposure and white-balance circuits. Any of these processes can produce optical fluctuations that are spatially coherent within an ROI and indistinguishable from a perfusion signal at the level of a single time series. The flap and a nonperfused area within the same field of view share these processes by construction. Their coherence values therefore covary, and the difference between them isolates what is unique to the flap.

### Why video-only signal separation matters

The absence of clinical covariate input is not incidental to the method; it is central to the present technical claim. AETHER does not know patient age, sex, body mass index, comorbidities, prior radiotherapy, smoking status, laboratory values, clinical suspicion or bedside examination findings. It receives only video. The observed case-level separation between ultimately vital flaps and flaps with surgically confirmed arterial compromise therefore cannot be explained by a multivariable risk model learning that older, irradiated, diabetic, or clinically suspicious patients are more likely to fail; nor can it be explained by leakage of clinician-provided labels into the input. The signal arises from the optical recording itself after correction for in-image common-mode artifacts. This strengthens the mechanistic interpretation of reference-corrected spatial coherence as a perfusion-associated optical structure that is detectable in routine video. It does not, on its own, establish clinical generalizability: Selection of the case-level cohort, the spectrum of compromise mechanisms present, and the absence of demographic effect-modification analyses remain limitations that only a prospective design can address.

### Why an interpretable signal-processing approach in a small pilot

This study intentionally uses interpretable signal processing rather than AI or deep learning. In a pilot cohort of 25 flaps with 6 events, training a black-box model would be a textbook example of insufficient data, and label-efficient representation-learning strategies that have been proposed for related medical-imaging tasks [19] still require many more samples than are available here. Interpretable signal processing supports transparent inspection of failure modes, allows the operator to reason about the corrected GCS, the absolute flap GCS, and the signal-to-noise ratio (SNR) of the raw traces jointly and remains computationally light enough for on-device inference on commodity smartphone hardware. AI-based components—automated segmentation, quality control, automated ROI selection, and temporal prediction—are valuable complements, and we identify them as future work. The choice is not framed as antagonistic to AI but as appropriate to the present sample size and to the deployment context.

### Comparison with established monitoring modalities

Each established free flap monitoring modality occupies a distinct point in the trade-off space. Clinical inspection (skin color, capillary refill, turgor, temperature, and pin prick) is universal, low-cost, and the established gold standard, but it is subjective, intermittent, and labor-intensive [6,7]. The implantable Cook–Swartz Doppler probe provides continuous pedicle-level signal but is invasive and has historically reported false-positive rates that limit its diagnostic specificity [8]; current devices and protocols achieve improved positive predictive value but still impose probe placement and removal as additional procedures. ICG angiography offers high spatial resolution and direct visualization of microvascular fill but is episodic and dye-dependent [9]. NIRS provides continuous regional oxygenation measurements with established clinical validity but requires dedicated hardware and adhesive sensors [10]. LDF continuously samples local microcirculation but is contact-dependent and prone to sensor detachment, particularly intraorally, and reports interpretable trends rather than absolute thresholds [11]. Imaging photoplethysmography and rPPG are contactless and impose minimal hardware burden but are exposed to optical confounders and superficial-signal limitations that any deployable system must contend with.

Within this landscape, AETHER occupies a different operating point. It is contactless, requires no consumables or device implantation, and runs on equipment already present at every postoperative bed. It is spatially resolved at 100 patches per flap, it is reference-corrected against the in-image artifact baseline, and the augmented overlay supports rapid visual interpretation. AETHER is therefore best positioned as a contactless, low-burden, spatially resolved spot-check adjunct, complementary to clinical inspection. It is not a replacement for established assessment; it does not provide continuous surveillance; and it does not interrogate the pedicle directly.

### Clinical positioning as a spot-check adjunct

As articulated by Creech and Miller (1975) and Swartz et al. (1988) and recently reiterated in the context of continuous LDF monitoring [11], the ideal flap monitoring system is continuous, distinguishes arterial from venous abnormalities, and is applicable to buried and cutaneous flaps. AETHER does not, in its current implementation, meet the continuity criterion. We state this without qualification. AETHER is a spot-check adjunct. It does not replace scheduled nursing checks. It does not detect events between acquisitions unless a recording is actively obtained. It does not reduce monitoring frequency or nursing workload, and it does not substitute for the intermittent clinical assessments that constitute the standard of care.

Its current value is the provision of objective, repeatable, spatially resolved quantitative documentation when a staff member is already present at the bedside and has elected to acquire a recording, and the support of decision-making in clinically ambiguous situations. Practical barriers to continuous or even frequent camera-based acquisition exist that are not solved by the current implementation: In breast (deep inferior epigastric perforator flap) reconstruction, axillary, groin, and perineal reconstructions the surgical site cannot remain continuously exposed. A continuous or semicontinuous camera-based extension would require dedicated hardware (fixed camera mount, illumination control, and automated ROI segmentation) and workflow validation; we frame this as a separate engineering program.

### Inherent limitations of camera-based rPPG monitoring

Several constraints are intrinsic to camera-based rPPG and must be made explicit. First, the green-channel rPPG signal has a shallow penetration depth of approximately 0.5 to 1.0 mm, interrogating principally the superficial dermis and papillary capillary loops. The superficial cutaneous signal does not directly represent deep flap perfusion or anastomotic patency; a normal corrected GCS does not exclude a deep problem, and an abnormal corrected GCS does not localize the lesion to the anastomosis. Second, in elderly patients, age-related dermal thinning and postirradiation skin changes make subcutaneous ecchymosis, hematoma, and edema more visually prominent; the superficial color signal may appear abnormal even when deeper tissue perfusion remains adequate, with a higher false-positive risk than in younger, unirradiated skin. PPG morphology is also modulated by vascular stiffness and is known to change with age [17]. Third, in musculocutaneous flaps, the skin paddle is supplied by perforators arising from the underlying muscle; when perforator caliber is insufficient relative to the skin island, the cutaneous component may exhibit transient color change and reduced turgor in the early postoperative period despite adequate muscular perfusion through the main pedicle. In such cases, the skin-paddle rPPG signal may underperform relative to the true viability of the flap. Fourth, partial compromise and heterogeneous perfusion are not fully addressed by a single manual flap ROI; a viable margin may rescue a partial lesion clinically while the AETHER coherence drops over the affected territory and rises elsewhere—full-surface coverage is required to detect such patterns reliably. Fifth, dressings, lighting drift, motion, exposure, camera model and operator handling all affect performance and were not standardized in this retrospective cohort. Finally, systemic variables (mean arterial pressure, heart rate, hemoglobin, vasopressor use, body temperature, and oxygen saturation) were not recorded and were not corrected for in the present analysis; their effect on PPG amplitude and morphology is well established and should be assessed in prospective work.

### Interpretation of negative corrected GCS values

Corrected GCS values reported for compromised flaps included negative values (IQR −2 to +13%, full range −6 to +17%). A negative corrected GCS indicates that the spatial coherence measured in the nonperfused reference ROI exceeds that measured in the flap ROI. Mechanistically, this can arise in 2 distinct scenarios with different clinical implications. First, low acquisition signal quality (suboptimal lighting, patient or camera motion, specular saturation, dressings within the flap ROI) may produce an artifactually low flap GCS, while the reference ROI, located on a more uniform optical surface, retains a normal artifact-driven coherence baseline. Second, severely compromised flap perfusion may suppress the flap pulsatile signal below the level of the simultaneously recorded optical artifact, so that the artifact baseline overwhelms any residual perfusion signal. The corrected GCS alone does not distinguish these scenarios, but both are clinically important: The first calls for repeat acquisition, the second calls for urgent surgical review.

We therefore recommend reporting the absolute flap GCS and an acquisition-quality signal-to-noise indicator alongside the corrected GCS. A low corrected GCS together with an interpretable raw SNR is more consistent with genuine compromise; a low corrected GCS together with poor raw SNR is more consistent with acquisition failure and warrants repeat acquisition before clinical action. Formalization of a confidence flag is future work. We note that the single false negative in this cohort (corrected GCS = +17%, flap GCS 46%, reference GCS 29%) had intermediate flap-GCS and SNR values and cannot be unambiguously attributed either to partially preserved pulsatility or to marginal acquisition signal to noise on the basis of the available data.

### Measurement duration and the case for patient-specific baselines

The present analysis is based on 10- to 22-s recordings; this is sufficient to characterize spatial phase coherence at a single time point but is too short to support heart-rate variability analysis or the kind of pulse-morphology feature extraction (area-under-curve, pulse width, rise time, skewness, kurtosis, and SNR) that requires minute-scale or longer acquisitions, as demonstrated in vitro by Ferizoli et al. [17]. The use of a single fixed threshold derived from such brief acquisitions across a heterogeneous flap population is of limited clinical value, and we frame the 17% cutoff exclusively as exploratory. Two complementary directions follow. The first is longer recordings supporting morphology and heart rate variability analysis. The second, supported by the intraoperative rPPG work of Schraven et al. [14], is the acquisition of a patient-specific baseline at the donor site prior to pedicle division—where perfusion is unequivocally normal—so that postoperative measurements can be referenced against the patient’s own baseline rather than against a population threshold.

### Prevalence dependence of PPV and NPV

PPV and NPV are functions of the underlying event prevalence. At the cohort prevalence of 24%, observed PPV and NPV were 0.71 and 0.94. At a more realistic background prevalence of 5%, applying the observed sensitivity (0.83) and specificity (0.89), the Bayes-projected PPV is approximately 0.29 and the NPV approximately 0.99. The system is therefore expected to act, in routine populations, principally as a rule-out test with high NPV and limited PPV. This is a structural property of the system at the present sensitivity and specificity. A clinically deployable workflow must account for prevalence: A positive AETHER finding in a low-prevalence ward setting requires clinical confirmation before acting; a negative AETHER finding has high (but not absolute) reassurance value.

### Methodological strengths

Three methodological choices warrant emphasis. First, the reference standard for compromise was surgical take-back with intraoperative confirmation of arterial compromise, performed by the operating team independently of the AETHER analysis; this avoids the circularity that affects studies in which the index test informs the reference standard. Second, the pipeline operates without manual signal grading, manual feature engineering, or operator-tunable thresholds beyond the initial ROI placement; once the 2 ROIs are defined, the corrected GCS is determined by the recording. Third, the analysis preserves the full distribution of per-patch and per-frame coherence values, which makes the visual signatures of vital and compromised flaps in the timeline output (decoupled versus coupled trajectories) detectable rather than collapsed into a single number. Fourth, the entire analysis is video-only: No clinical covariate is supplied to the algorithm, so the discriminative signal is a property of the recording itself and cannot be attributed to hidden confounding through clinical metadata.

### Limitations

This study has substantial limitations. (a) The cohort is small (*n* = 25), single-center, and retrospective; 6 events produce wide Clopper–Pearson intervals around the sensitivity point estimate, and the cutoff of corrected GCS = 17% was identified by Youden optimization on the same cohort and remains an exploratory working threshold until prospectively validated. (b) The 24% within-cohort event proportion reflects enrichment of cases with available bedside video documentation; it is not the institutional free-flap compromise rate, and PPV and NPV at this prevalence are not generalizable. (c) Because the anonymized analytic dataset did not include patient-level demographic, comorbidity, and temporal clinical metadata, we cannot assess whether age, sex, skin type, irradiation, comorbidities, or systemic hemodynamic variables modify performance; we cannot estimate the exact postoperative hour at which a recording was obtained relative to the onset of compromise; and we cannot infer lead time, alarm performance, or whether compromise was detectable at the time of recording. Consequently, the present analysis cannot constitute recording-level diagnostic validation. We therefore frame the AUC as an exploratory measure of case-level signal separation between ultimately vital flaps and flaps with surgically confirmed arterial compromise, not as recording-level diagnostic performance. (d) Manual ROI selection is operator-dependent; inter- and intrarater reproducibility have not been formally measured. (e) The control group includes only flaps with uncomplicated postoperative course; clinically suspicious but ultimately viable flaps, transient congestion, partial compromise not requiring revision, and false-positive clinical alarms are not represented, and the resulting spectrum bias is likely to inflate the apparent AUC relative to realistic clinical performance. (f) All 6 compromised cases had arterial compromise; AETHER has not yet been characterized for venous congestion, partial flap compromise, or subsegmental ischemia. (g) Performance across other flap types (deep inferior epigastric perforator flap, latissimus, radial forearm, fibula, and jejunum), across the full Fitzpatrick I to VI skin-tone range, in pediatric anatomy, and in nonstandard lighting has not been characterized; deeply pigmented skin is known to attenuate the chromatic component on which plane-orthogonal-to-skin-based pulse extraction relies. (h) The analysis is currently performed offline on completed recordings rather than continuously in real time at the bedside.

Despite these limitations, the study provides evidence that reference-corrected spatial coherence can extract a perfusion-associated signal from routine video alone, without input from clinical covariates. AETHER should not be used for clinical decision-making until prospectively validated and integrated into a predefined monitoring protocol that includes temporal metadata, clinical covariates, venous and partial compromise, suspicious-but-viable cases, and a locked decision threshold.

### Outlook and future work

Five concrete directions follow from this pilot. (a) A prospective multicenter validation study with a prespecified locked corrected-GCS cutoff and a standardized acquisition protocol, collecting patient-level demographic, comorbidity, and systemic-hemodynamic variables alongside the video signal. With an anticipated event rate of 4% to 8%, a cohort on the order of 200 to 400 flaps would provide tight confidence intervals around sensitivity and specificity. The validation cohort must include venous congestion, partial flap compromise, subsegmental ischemia, clinically suspicious but ultimately viable flaps, and transient congestion, none of which are represented in the present arterial-compromise pilot. (b) Head-to-head comparison against clinical inspection by experienced and inexperienced observers, implantable Doppler, ICG angiography, NIRS, and LDF in the same cohort, to quantify lead-time benefit and false-alarm trade-offs. (c) Methodological extensions, including automated semantic segmentation of flap and reference and full-surface coverage of the flap rather than a single bounding ROI—for which recent computational biomedical image-analysis methods such as spatial–spectral fusion segmentation and multiscale window transformers are directly relevant [20,21]—together with inter- and intrarater reproducibility testing, confidence flags for individual recordings to identify the gray zone before clinical interpretation, real-time on-device implementation, and characterization of the visual signatures associated with venous congestion and partial compromise. (d) Acquisition of a patient-specific baseline at the donor site prior to pedicle division, allowing postoperative measurements to be referenced against the patient’s own perfused-tissue signal rather than against a population threshold [14]. (e) Longer recordings supporting heart-rate variability and pulse-morphology feature analysis [17], together with prospective recording of systemic covariates (mean arterial pressure, heart rate, hemoglobin, vasopressors, temperature, and oxygenation) for multivariable adjustment. Integration of AETHER outputs with clinical covariates in a multivariable prediction model is a logical next step beyond the present video-only proof of concept.

## Conclusions

In this retrospective video-only proof-of-concept study of 25 ALT free flaps, AETHER demonstrated strong in-sample case-level separation between ultimately vital flaps and flaps with surgically confirmed arterial compromise using only reference-corrected spatial coherence from routine smartphone video. No patient-level demographic, comorbidity, clinical-suspicion or bedside-examination data entered the algorithm; the observed separation is a property of the optical recording after correction for in-image common-mode artifacts. Because temporal clinical metadata and patient-level covariates were not available, these findings should be interpreted as evidence of a video-derived perfusion-associated signal in ALT flaps with surgically confirmed arterial compromise, not as validated diagnostic performance and not as evidence of generalizability to venous congestion, partial compromise, or other flap types. AETHER is positioned as a contactless spot-check adjunct, not as continuous monitoring, and should not be used for clinical decision-making until prospectively validated in a predefined monitoring protocol that includes temporal metadata, clinical covariates, venous and partial compromise, and suspicious-but-viable cases. Within these constraints, the present results support the broader claim that interpretable perfusion-associated information is recoverable from routine bedside video using equipment that already exists at every postoperative bed and that disciplined reference correction is essential before any such signal can be trusted as a perfusion marker.

## Ethical Approval

This retrospective, single-center observational study was conducted in accordance with the Declaration of Helsinki and approved by the institutional review board of the Ärztekammer Westfalen-Lippe (approval no. 2026-037-f-S). Due to the retrospective design and the exclusive use of fully anonymized routine clinical data, the requirement for informed consent was waived by the ethics committee.

## Data Availability

The data that support the findings of this study are not publicly available due to institutional data protection requirements and patient privacy regulations. Deidentified data and per-recording corrected GCS values may be made available from the corresponding author upon reasonable request and subject to approval by the local ethics committee and institutional governance. Table 1 provides per-recording flap GCS, reference GCS, and corrected GCS values.
